# Esophageal Atresia: Migration of the gastrostomy tube into the bronchus

**DOI:** 10.4103/0971-9261.43823

**Published:** 2008

**Authors:** Seyed Mohammad Vahid Hosseini, Seyed Abbas Banani, Babak Sabet, Sam Zeraatian, Tannaz Razmi, Seyed Javad Banani

**Affiliations:** Department of Pediatric Surgery, Shiraz University of Medical Sciences, Shiraz, Iran

**Keywords:** Esophageal atresia, gasless abdomen, gastrostomy tube, surgical complication

## Abstract

A 2-day-old baby boy, 38 weeks gestation, weight 2000 g was brought due to hypersalivation and imperforate anus with gasless abdomen on plain X-ray. He underwent a gastrostomy tube insertion and colostomy. In contrast study of the stomach, on the 5th postoperative day, the dye spilled into the tracheo bronchial tree and the catheter was seen, entering the right main bronchus. The patient underwent right thoracotomy and the presence of fistula and catheter were confirmed. The fistula and distal esophagus were closed and fixed to the prevertebral fascia because of a long gap. He is under follow-up and recieving home care for a later delayed primary anastomosis.

## INTRODUCTION

The gastrostomy tube (G tube) has been the main part in the treatment of many pediatric surgical problems, but advances in neonatal care and surgical techniques[1] have made its usefulness to specific circumstances like pure esophageal atresia (EA), staged operation in EA and gastroesophageal reflux, for decompression and feeding. There is no difference in the type of complications in pediatric G tube and those that were inserted in adult patients. Although G tube insertion is not a difficult procedure, precise postoperative care should be counselled to prevent G tube complications.[2]

## CASE REPORT

**Figure 1:**
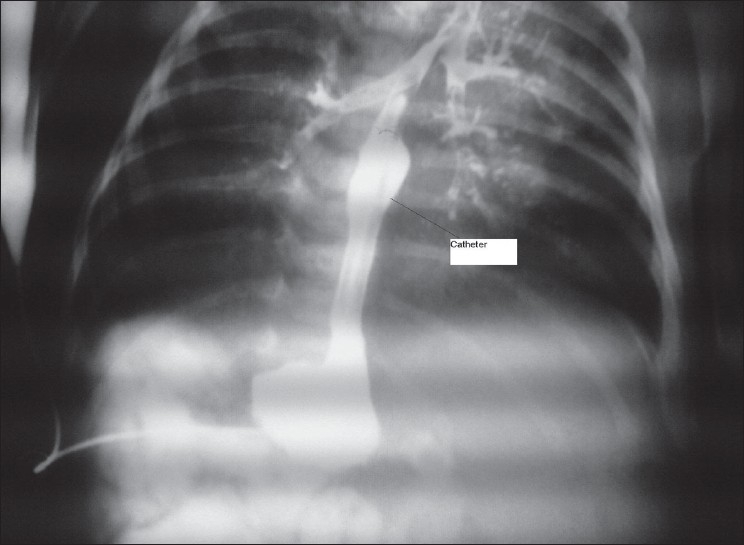
X-ray shows spillage of contrast material and tube entering bronchus

A 2-day-old boy, G/A 38 weeks, weight 2000 g, was brought due to hypersalivation and imperforate anus. Because of gasless abdomen (pure EA) on plain X-ray, situs inversus and high type imperforate anus; he underwent a 8 Fr Foley catheter gastrostomy and colostomy. He had a small and diffuse stomach at the time of G tube insertion. After 5 days, he underwent a water soluble contrast study through G tube but the X-ray result showed migration of the G tube into the right main stem bronchus and spillage of contrast into the tracheobronchial tree [Figure 1]. He developed some respiratory distress and, after stabilization, had right thoracotomy for fistula closure and esophageal repair.

**Figure 2:**
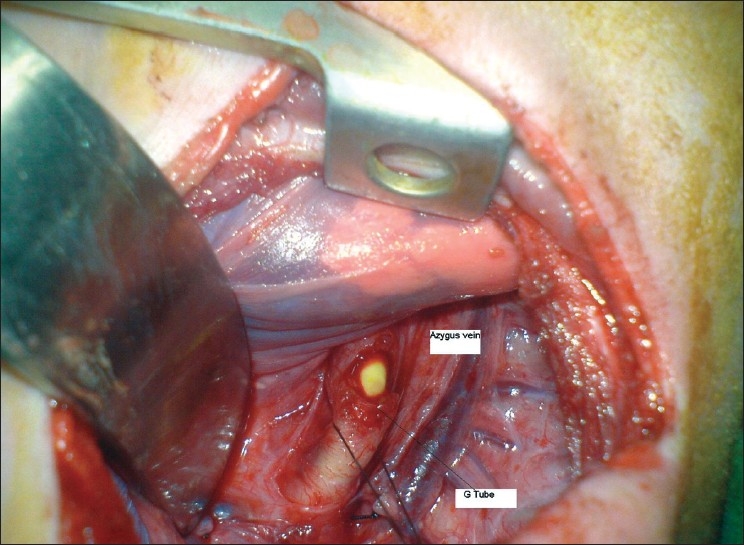
Intraoperative finding of G tube in fistula

The intraoperative finding [Figure 2] was a distal fistula with a proximal 5–7 mm cartilaginous part containing the Foley catheter that entered into the right main bronchus after division and closure of the fistula. The proximal part was not well developed and the proximal cartilaginous segment of the distal part had been resected; therefore, two ends had more than a five vertebral bodies gap. The distal segment was overclosed with PDS 5/0 and fixed with a nonabsorbable stitch to the prevertebral fascia at the level of the diaphragm. He had a good postoperative recovery and is being followed-up for delayed primary anastomosis.

## DISCUSSION

G tube is one of the oldest surgical procedures that have been performed for by-passing the upper aero digestive tract, for feeding or decompression of the stomach. It has been used in pediatric patients with cerebral palsy, gastroesophageal reflux, tracheoesophageal fistula, etc.[1,2]

G tube is usually inserted by a surgical procedure through an opening that is created between the gastric mucosa and the abdominal wall or a percutaneous insertion with the guidance of an endoscope or under fluoroscopic control. G tube has three parts, which include the internal part, the external part and the feeding port,[3,4] and different surgical tubes have been used for insertion as G tube (mush room catheter, Foley catheter, etc.) such that their special cares prevent further problems in G tube usage.

Complications of G tube (1–14%) include those related to surgical procedures, like wound infection, intraabdominal leak and pnemoperitonium (35%) and the problems related to gastrostomy creation, e.g. periostomal dermatitis, bleeding, granuloma and gastroesophageal reflux. Surgical complications usually occur in the first few postoperative days, which should be evaluated by contrast studies; however, many of them would improve with conservative management. Minor complications like tube migration and secondary gastrointestinal obstruction or tube blockage can be prevented by good nursing care including, repeated skin length measuring, checking balloon function and tube irrigation.[5–9]

According to our knowledge, this is the first case in which the G tube has migrated retrograde and so high that it has entered the right main bronchus. Its migration can be explained by repeated manipulation and the constant negative pressure that was exerted through the fistula associated with balloon deflation. We conclude that if the tube is checked constantly for position, length from skin and balloon functioning, majority of these problem can be prevented, which lessens not only the psychic impact on parents and patients but also gives the chance of better G tube performance.
